# Anisakiasis in the Upper Esophagus: A Case Report

**DOI:** 10.3390/medicina59101888

**Published:** 2023-10-23

**Authors:** Eiji Kamba, Takashi Murakami, Hiroya Ueyama, Tomoyoshi Shibuya, Mariko Hojo, Ken Yamaji, Akihito Nagahara

**Affiliations:** 1 Department of Gastroenterology, Juntendo University School of Medicine, Tokyo 113-842, Japan; 2 Department of Internal Medicine and Rheumatology, Juntendo University School of Medicine, Tokyo 113-842, Japan

**Keywords:** *Anisakis* sp., esophageal anisakiasis, parasitic infection, precordial pain

## Abstract

Anisakiasis is caused by consuming raw fish contaminated with *Anisakis* sp. larvae and is extremely rare, especially when originating in the esophagus. We present a case of esophageal anisakiasis in a 61-year-old male who experienced severe precordial pain and radiating discomfort to the neck after consuming raw fish sashimi. Upper gastrointestinal endoscopy revealed the presence of a larva in the upper esophagus. On the basis of anatomo-morphological features, the worm was provisionally identified as *Anisakis* sp. and was easily extracted with forceps, which led to a prompt improvement in the patient’s symptoms. This case highlights the importance of considering anisakiasis as a differential diagnosis in patients with gastrointestinal symptoms and a history of consuming raw fish.

## 1. Introduction

Anisakiasis is a parasitic gastrointestinal disease that causes various gastrointestinal symptoms and can occur after the consumption of raw or undercooked seafood infected with *Anisakis* sp. larvae. Patients’ symptoms range from mild abdominal pain to severe complications, such as a bowel obstruction or perforation. Reports of this disease are frequent in Japan, where the consumption of raw seafood is common. However, most cases originate in the stomach, while esophageal anisakiasis is extremely rare [1]. We describe a case of esophageal anisakiasis caused by the penetration of an *Anisakis* sp. larva in the upper esophagus of a patient.

## 2. Case Presentation

**Figure 1:**
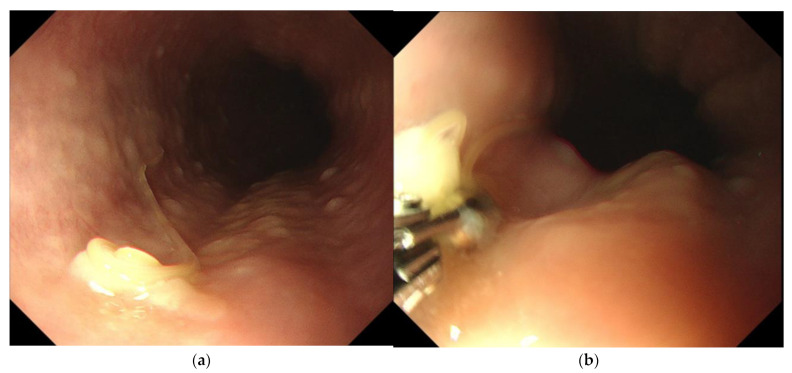
(**a**) Esophagogastroduodenoscopy image showing an *Anisakis* sp. larvae invading the mucosa of the upper esophagus. (**b**) Esophagogastroduodenoscopy image showing the removal the worm with biopsy forceps.

A 61-year-old male developed severe precordial pain 8 h after consuming raw fish sashimi (consisting of *Lateolabrax japonicus*, *Salmo salar*, *Seriola quinqueradiata*, and *Lutjanus stellatus*). The patient experienced a gradual increase in radiating pain to both sides of the neck, prompting him to visit the emergency department of our hospital the next day. The patient had no significant medical history or known allergies. He was 175 cm tall and had a healthy weight of 65 kg. A physical examination revealed a body temperature of 36.6 °C, blood pressure of 146/87 mmHg, a respiratory rate of 12 breaths per minute, an SpO₂ value (room air) of 99%, and was alert. Upon the auscultation of the chest, breathing sounds were found to be normal, no significant heart murmurs were detected, and no spontaneous or palpable pain was observed in the upper abdomen. An electrocardiogram did not reveal any signs of ischemic heart disease. We subsequently suspected anisakiasis based on the patient’s history of consuming raw fish and performed an esophagogastroduodenoscopy (EGD) using a GIF-H290 videogastroscope (Olympus, Tokyo, Japan) on the same day. The EGD revealed the presence of a worm-like structure, approximately 20 mm in length, embedded in the mucosa of the upper esophagus, approximately 25 cm from the incisors (Figure 1a). On the basis of anatomo-morphological features, the worm was provisionally identified as a third-stage *Anisakis* sp. larva by two gastroenterologists at Juntendo University (Eiji Kamba and Takashi Murakami). The molecular identification of the nematode was not performed. Acute mucosal lesions, other than the *Anisakis* sp. larva, or vanishing tumors were not found in the rest of the esophagus, stomach, and duodenum. The *Anisakis* sp. larva was swiftly and easily removed using biopsy forceps (Figure 1b) and the patient’s symptoms promptly improved to the point that within 15 h they had disappeared. Subsequently, the patient made a complete recovery.

## 3. Discussion

Gastrointestinal anisakiasis is a zoonotic parasitic infection resulting from the ingestion of Anisakis sp. nematodes present in raw or undercooked seafood. The disease was first reported by van Thiel et al. in 1960 [2]. Marine mammals, including cetaceans, Eumetopias jubatus, members of the Phocidae family, Delphinus sp., harbor porpoises, and Odobenus rosmarus, are recognized as the primary hosts of Anisakis sp., while humans are considered incidental hosts [3]. It is noteworthy that these parasites are incapable of developing into adult nematodes within humans. The life cycle is initiated when mature nematodes within their natural host excrete unembryonated eggs in their fecal matter [4]. The eggs undergo embryonation, subsequently yielding first- and second-stage larvae that are subsequently released as free-living second-stage larvae.

The second-stage larvae are ingested by crustaceans (intermediate hosts) in which they develop into third-stage larvae. These can be passed on to fish and squid, and become infectious to humans when accidentally ingested [5]. Salmonidae, Clupea pallasii, Gadus morhua, Scomber, and Decapodiformes are well-known intermediate hosts that can be infected with third-stage larvae. Live Anisakis simplex third-stage larvae penetrate the gastrointestinal mucosa after they are ingested in raw or undercooked seafood, consequently causing gastrointestinal symptoms and allergies. Previous reports described the Anisakis sp. larvae as displaying a cylindrical body shape, tapered at both ends, and with dimensions of 14.1–25.6 mm in length (a mean of 20.4 ± 1.2 mm) and 0.48–0.62 mm in width (a mean of 0.55 ± 0.02 mm) [6,7]. Microscopically, such larvae are enveloped by a rigid cuticle featuring circular transverse striations that commence from the cephalic region and extend toward the anus and which have a slit-shaped appearance. The lips are not easily discernible but a prominent boring tooth is located at the front end. Furthermore, longitudinal lateral grooves run along the larval body, originating near the mouth area and concluding before the mucron [8].

Currently, nine known species exist within the Anisakis sp. genus, and their third-stage larvae are classified into four types, namely, Types I to IV, based on morphological differences [9]. Of these nine Anisakis sp., six, namely, Anisakis simplex sensu stricto, A. pegreffii, A. berlandi, A. typica, A. ziphidarum, and A. nascettii, are classified as Type I larvae. Morphologically, such larvae are characterized by a body length/stomach length ratio of 26.4 ± 2.3, with well-developed stomachs compared to Types II to IV larvae, and can be readily observed with the naked eye. However, the morphological identification of these six Type I larvae can be challenging. Therefore, species are generally identified by sequencing the ribosomal RNA gene internal transcribed spacer (ITS) region, which spans 930 to 957 base pairs [10]. In rare instances, anisakis hybrids, referred to as hybrid types, have been reported to possess sequences of both A. simplex and A. pegreffii at the 5’ end of ITS1, making differentiation between the two species difficult based solely on ITS region sequencing results [11]. In such cases, identification is performed through the phylogenetic analysis of the cytochrome c oxidase subunit 2 gene of the mitochondrial genome, which encodes a 629-base pair sequence. In comparison, Types II, III, and IV larvae can be identified based on their respective morphologies as A. physeteris, A. brevispiculata, and A. paggiae, respectively.

While gastrointestinal anisakiasis has been extensively reported in Japan, where a tradition of consuming raw seafood exists, the consumption of raw fish has increased in Western nations and caused a rise in the incidence of clinical cases of anisakiasis [12,13]. The Ministry of Health, Labour and Welfare’s Food Poisoning Statistics Survey in Japan recorded 396 cases of anisakiasis in 2020, 354 cases in 2021, and 578 cases in 2022, revealing an increasing trend. However, data based on prescription records estimate an annual incidence of 7000 cases [14]. Furthermore, in food poisoning statistics from 2013 to 2021, the age distribution data of patients with anisakiasis revealed that, at 26.3%, the majority were in their 40s followed by those in their 30s at 23.7%. The pediatric population, specifically those under 15 years of age, accounted for only 0.4% of total patients, indicating a minority of cases in this age group [15].

Anisakiasis can be categorized into three distinct types: gastric, intestinal, and ectopic [13]. The most prevalent form is gastric anisakiasis, constituting approximately 95% of all reported cases. Intestinal anisakiasis represents the majority of the remaining cases, while the ectopic subtype, also known as extragastrointestinal anisakiasis, is an exceedingly rare condition [13]; several reports exist in the English medical literature [16,17,18,19,20], but only two cases were submitted to PubMed [21,22].

The clinical manifestations of anisakiasis are not only confined to gastrointestinal symptoms, such as nausea, vomiting, and epigastric pain, but also include allergic reactions to the penetration of Anisakis sp. larvae into the gastrointestinal wall, including angioedema and urticaria. It is common for the condition to be accompanied by the presence of ascites or symptoms indicative of peritoneal irritation. In cases of small intestinal anisakiasis, the condition can also lead to symptoms of intestinal obstruction or intestinal intussusception [23]. From a serological diagnostic perspective, an increase in the white blood cell count and elevated serum levels of C-reactive protein, along with the measurement of serum Anisakis sp.-specific IgG and IgA antibodies, can serve as useful references. However, it is important to note that the detection rate for these antibodies is approximately 70–80%, and false negatives can occur, especially in the immediate aftermath of symptom onset [24]. Therefore, the use of paired sera is recommended for antibody measurements for greater diagnostic accuracy. Acute symptoms, such as nausea, vomiting, epigastric pain, and low-grade fever, typically begin from 2 to 12 h after consuming infected seafood. Asymptomatic cases have been identified incidentally. Esophageal anisakiasis often manifests as ischemic cardiac-like symptoms, including discomfort in the thoracic and dorsal regions, as reported by Adachi et al. [25]. Therefore, if anisakiasis is suspected, carefully questioning patients about their dietary history is essential if a diagnosis is to be made [16,26,27]. Furthermore, anisakiasis is known to be a factor contributing to systemic anaphylaxis. Anaphylactic reactions to Anisakis sp. are postulated as one of the causes of so-called food allergies [28]. In addition, in intestinal anisakiasis, larvae penetrate the intestinal wall. Histologically, this condition can be broadly categorized into two main types: cellulitis characterized by eosinophil and lymphocyte infiltration extending from the intestinal wall where the parasite has penetrated to the serosa; or granuloma formation centered around the remnants of dead parasites within the tissue. Clinically, anisakiasis can be classified into acute and subacute types, each of which closely corresponds to cellulitis and granuloma formation, respectively [29]. The majority of clinically diagnosed cases of intestinal anisakiasis are of the acute type. The immunological mechanisms underlying cellulitis involve types I or III allergic reactions triggered by the penetration of the parasite. Therefore, when a Anisakis sp. parasite penetrates the intestinal wall in individuals who have not been previously sensitized, they develop and recover from the condition without developing intestinal anisakiasis. One of the antigens of Anisakis sp., Ani s1, has been isolated, which specifically reacts with patient IgE. However, cases exist where anaphylaxis occurs without accompanying intestinal anisakiasis, suggesting that the immune response to Anisakis sp. may vary depending on factors such as the type of antigen, individual differences, or the specific organ affected.

In the context of gastric anisakiasis, diagnosing the condition using endoscopic imaging is relatively straightforward since whitish, thread-like, worm-like bodies can be observed. Apart from local redness, edema, and ulceration, the presence of such worm-like bodies can cause inflammation in the submucosal layer, resulting in a tumor-like elevation. This elevation, often referred to as a “vanishing tumor”, tends to resolve within approximately 1 to 2 weeks along with an improvement in the inflammation. Approximately 2–4% of gastric anisakiasis cases present with a vanishing tumor [30]. In some instances, the worm-like bodies may not be visible during endoscopy. When such worm-like bodies are not observed, and significant mucosal folding and swelling is observed in the stomach, the differential diagnosis may include diffuse infiltrating (Type 4) gastric cancer, acute gastric mucosal lesions, giant rugal fold hypertrophic gastritis, corrosive gastritis, gastric cellulitis, or gastric syphilis, among other causes. In cases of gastric anisakiasis, mucosal folding and swelling in the stomach often resolve within 1 to 2 weeks. Therefore, if mucosal folding and swelling are observed, but cancer is not found in a biopsy, a repeat endoscopic examination within a short period of time should be considered to distinguish this condition. Reports exist of cases in which gastric cancer was initially suspected in conjunction with gastric anisakiasis; however, prior to surgery, the mucosal folding and swelling had disappeared [31]. In such cases, a more limited resection (such as a pylorus-preserving gastrectomy) was performed instead of a total gastrectomy to avoid excessive treatment. Pathologically, diffuse eosinophilic infiltration, edema, and fibrosis deposition are evident, particularly in the submucosal layer of the entire gastric wall [32]. Additionally, mild vascular and lymphatic dilation, along with minor hemorrhage, can also be observed. These reactions are believed to be a result of an Arthus-type (Type III) allergic response. Furthermore, within the gastric wall, especially in the submucosal layer, the presence of parasites or their keratinized forms may be noted. In some instances, this may lead to the formation of parasitic or eosinophilic granulomas, with such structures occasionally located at their centers. When encountering significant eosinophilic infiltration in the deep layers of the mucosa during a biopsy, particularly from gastric submucosal tumor-like elevations, the presence of Anisakis sp. should be suspected, even if no actual parasites are detected.

Regarding the mechanism of Anisakis sp. larvae penetrating the esophagus, Urita et al. postulated that larvae initially entered the stomach and then returned to the esophagus by gastroesophageal reflux, leading to esophageal anisakiasis [22]. However, since an esophageal hernia was not observed in this case, we are somewhat skeptical that an Anisakis sp. larva was regurgitated from the stomach into the upper esophagus. It is possible that esophageal penetration was caused by food stasis, but the true mechanism currently remains unknown.

The mainstay of treatment is an early endoscopic extraction. It is important to grab a larva as close to its embedded portion as possible to ensure no part of the larva remains within the mucosa. If not removed completely, the larva can cause chronic inflammation leading to various gastrointestinal symptoms. Conservative treatment with anti-allergic medication is considered effective against anisakiasis. However, it should be noted that the presence of invading larvae can lead to granuloma formation [33]. Additionally, several reports exist of a case accompanied by mediastinitis, indicating the possibility of perforation in esophageal anisakiasis due to the thinner esophageal wall compared to the gastric wall [34]. Therefore, if anisakiasis is suspected, a prompt upper gastrointestinal endoscopy should be considered. Various techniques exist to enhance seafood safety and reducing the risk of human anisakiasis, such as freezing, heating, high hydrostatic pressure, salting, pepsin digestion, and the use of garlic oil. These techniques have shown some effectiveness in different studies and may be cost-effective strategies, either used alone or in combination, to reduce the risk of anisakiasis. However, recommendations for seafood safety are limited due to the diversity of seafood and the specific requirements for inactivating Anisakis sp. larvae. Further research is needed to determine which method or combination of methods is most effective in killing Anisakis sp. larvae in seafood products, particularly in raw fish products, such as sushi and sashimi [35]. In this case, the immediate endoscopic removal of the Anisakis sp. larva in the upper esophagus resulted in the complete resolution of the patient’s symptoms.

## 4. Conclusions

Although esophageal anisakiasis is an extremely rare condition, it should be considered during the differential diagnosis of patients presenting with gastrointestinal symptoms and a history of consuming raw fish. Obtaining a detailed food history will often be the key to a diagnosis as symptoms usually arise shortly after the ingestion of food contaminated with the parasite.

## Data Availability

This data is available on PubMed and Google Schoolar.
